# Use of Heating Methods and Xylose to Increase Rumen Undegradable Protein of Alternative Protein Sources: 2) Cottonseed Meal

**DOI:** 10.3390/ani13010041

**Published:** 2022-12-22

**Authors:** Vitor L. Molosse, David A. B. Pereira, Fernanda Rigon, Kalista E. Loregian, Elaine Magnani, Marcos I. Marcondes, Renata H. Branco, Pedro D. B. Benedeti, Eduardo M. Paula

**Affiliations:** 1 Department of Animal Science, Universidade do Estado de Santa Catarina, Chapecó 89815-630, SC, Brazil; 2 Instituto de Zootecnia, Centro APTA Bovinos de Corte, Sertãozinho 14160-970, SP, Brazil; 3 Department of Animal Sciences, Washington State University, Pullman, WA 99164, USA

**Keywords:** beef cattle, ruminal degradation, protein degradation, protein feedstuff

## Abstract

**Simple Summary:**

Scientists constantly seek techniques that may improve animal-feed usage, because they can reduce environmental impact and increase the profitability of feedlot systems. In beef-cattle production systems, high-performance animals need a greater protein supply that escapes rumen fermentation to be digested in the intestine compared to low-performance animals. This fraction is called rumen undegradable protein (RUP). Cottonseed meal is a possible protein source with an excellent amino-acid profile; however, cottonseed meal has low RUP content, which can be increased by applying heat and xylose. Thus, we submitted this feed to different heat techniques (autoclave, conventional, and microwave ovens), with and without xylose treatment, to increase its RUP content. Our results suggest that the evaluated processing methods may increase cottonseed meal RUP. The best treatments under the experimental conditions were: for the autoclave, xylose-treated cottonseed meal with 8 and 16 min heating; for conventional oven, 90 min heating for xylose-treated cottonseed meal; and for the microwave oven, xylose-treated cottonseed meals with 2, 4, and 6 min heating. Further studies are necessary to confirm the results found here and evaluate the effects of these processed ingredients on ruminal fermentation parameters, animal performance, and economic viability.

**Abstract:**

The ruminal kinetics of protein sources may be changed by heat and sugar treatments. Thus, these processing methods may be used as alternatives to increase beef-cattle diets’ rumen undegradable protein (RUP). We aimed to evaluate the effects of processing cottonseed meals with autoclave, conventional, and microwave ovens, with and without using xylose, on the ruminal kinetics degradation parameters and intestinal digestibility (ID). In situ studies were conducted, and each sample was incubated in the rumen to determine dry matter (DM) and crude protein (CP) rumen degradation kinetics. In vitro studies were also conducted to evaluate ID. The control treatment had a greater soluble fraction for DM and CP than processed cottonseed meals (*p* < 0.05). The addition of xylose decreased both DM and CP water-soluble fractions (fraction A) of cottonseed meal heated in a conventional oven (*p* < 0.05). Compared to the control, we observed a decrease in effective degradability and increased RUP for all processed methods (*p* < 0.05). Furthermore, conventional and microwave ovens showed greater ID than the control. Moreover, xylose-treated groups heated in the autoclave and conventional ovens had greater ID than xylose-untreated cottonseed meal. Under these experimental conditions, cottonseed RUP was increased by the evaluated processing methods.

## 1. Introduction

Soybean meal is the primary protein source in Brazilian beef-cattle feedlot diets [1]. However, beef nutritionists have been seeking alternative protein sources to reduce feeding costs due to their high prices. As a result, cottonseed meal has been adopted as an alternative to soybean meal, not only as a protein source but also as a good source of metabolizable energy and a lower price [1,2]. Therefore, using cottonseed meal appears to be an affordable strategy to provide a good protein source and reduce feeding costs. However, the protein proportion of cottonseed meal that escapes ruminal fermentation can be as low as 24% of total protein depending on its degradation rate and oil extraction procedures [3,4]. In an in vitro study, Broderik (1980) [4] observed that the crude protein (CP) ruminal degradation of solvent-extracted cottonseed meal varied from 21.2 to 44.4%. Another study conducted by Sadeghy (2007) [3] observed effective rumen degradations of 76.0, 61.0, and 52.9% for untreated cottonseed meals with rumen outflow rates of 0.02, 0.05, and 0.08/h, respectively. Thus, to reach rumen undegradable protein (RUP) requirements of high-performance animals may be difficult when cottonseed meal is used as the main protein source.

High-performance animals have higher RUP requirements than low-performance animals [1,5]. For instance, the RUP/CP ratio requirement is 0.528 for a growing bull averaging 430 kg of body weight and 1.95 of average daily gain [2]. Thus, improvement of feed’s RUP could improve nitrogen-utilization efficiency and, consequently, the muscle-mass gain of beef cattle. Furthermore, from an environmental standpoint, higher efficiency could decrease nitrogen excretion and greenhouse emissions, making the system more sustainable [6]. Therefore, processing techniques that improve RUP may allow high-producing animals to express their potential performance [7].

Different processing methods, such as heating and xylose treatment [8] can change ruminal degradation of feed [8]. For example, heat treatment may lead to a browning reaction by binding the carbonyl group of reducing sugars with the amino group of proteins (Maillard reaction), which might increase feed RUP content [9,10]. Furthermore, xylose (a reducing sugar) may also contribute to heating to decrease ruminal degradation by catalyzing the reaction between the aldehyde groups and amino acids [11,12]. However, it is essential that this bond can be reverted by the low pH of the abomasum so that protein can be digested and absorbed in the intestine [11,13]. Insoluble polymers are formed during the final stages of the Maillard reaction if samples are overheated, decreasing intestinal digestibility [8]. As such, the time and intensity of heat exposure may affect amino-acid availability for intestinal absorption [14,15].

Autoclaves [16], toasting [17], and microwave [3,18,19] have been studied as methods to improve the RUP of protein sources. However, there is little information in the literature about the consequences of applying these processing methods and xylose treatment on ruminal degradation parameters of cottonseed meal [20,21]. Sacakli (2006) [8] did not observe effects of water plus heat (100 °C) treatment, with and without xylose inclusion (10 g/kg DM), on cottonseed meal protein protection. According to these authors, higher dosages of xylose would be required to optimize Maillard reaction effects on cottonseed meal. On the other hand, Sadeghi and Shawrang (2007) [3] observed a digestible undegraded protein increase when cottonseed meal was microwaved for 4 min, but they did not test the interaction with xylose. Therefore, this study evaluated the effects of processing cottonseed meal with autoclave, conventional, and microwave ovens, with and without using xylose, on ruminal kinetics degradation parameters and intestinal digestibility (ID). We hypothesized that processing methods would change ruminal parameters and increase RUP without affecting ID. A companion paper evaluated the same processing methods for increasing the RUP of peanut meal [22].

## 2. Materials and Methods

### 2.1. Location, Heating Processing Methods, and Chemical Analysis

This is one of two companion papers that evaluated heating processes and xylose to increase RUP of different protein sources (cottonseed and peanut meals). All experiments were conducted at the Instituto de Zootecnia, Beef Cattle Research Center, Sertãozinho, São Paulo, Brazil. The meal used in this study resulted from physical (pressing) and chemical (using hexane as solvent) oil extractions from cottonseed seed. Three experiments were performed for evaluation of processing methods of cottonseed meal: autoclave (Experiment 1, Table 1), conventional oven (Experiment 2, Table 2), and microwave oven (Experiment 3, Table 3). The design and all procedures were the same for the three experiments. Therefore, seven treatments were investigated within each experiment: control (feed without xylose and heat processing) and three heating times applied to both xylose-treated (inclusion of 20 g/kg DM) and -untreated cottonseed meals. The xylose was obtained from Sigma-Aldrich^®^ with ≥99% purity. The heating times were 8, 16, and 24 min for the autoclave (127 °C and 117 kpa of pressure); 30, 60, and 90 min for the conventional oven (150 °C); and 2, 4, and 6 min for the microwave oven (1000 W, full power).

All ingredients used in these studies were ground through a 2 mm screen (Wiley mill; Thomson Scientific Inc., Philadelphia, PA, USA) for performing all incubations and analysis. Samples were analyzed for dry matter (DM; method G-003/1), ash (method M-001/1), crude protein (method N-001/1), and ether extract (EE; method G-005/1) according to Detman et al. (2012) [23]. Organic matter (OM) was calculated as the difference between DM and ash contents. For neutral detergent fiber (NDF), samples were treated with alpha thermo-stable amylase omitting sodium sulfite [24], and adapted for the Ankom^200^ Fiber Analyzer (Ankom Technology, Macedon, NY, USA). Chemical composition of experimental ingredients is presented in Table 1, Table 2, and Table 3 for Experiment 1 (autoclave), Experiment 2 (conventional oven), and Experiment 3 (microwave oven), respectively.

### 2.2. In Situ Procedures and Calculations

Regarding in situ evaluation, a set of three different cannulated Nellore steers (n = 9, total, average BW of 397 ± 51 kg) was used for each experiment. Animals were housed in an enclosed barn, restrained in individual tie stalls, and fed a 60:40 forage to concentrate diet (60% corn silage, 24.9% dry ground corn, 13% soybean meal, 0.2% urea, and 1.9% mineral mixture). Steers were adapted to this diet 14 d before the study; they had free access to feed and water. Ingredients were individually weighed into nylon bags (Ankom R510; 50 μm porosity, 400 cm^2^ surface area) and incubated in each animal. The bag surface area to mass ratio was 15 mg/cm^2^. For each ingredient, simultaneously in each steer, bags were incubated in the rumen for 2, 4, 8, 12, 24, and 48 h. Samples within filter bags were incubated for each treatment in triplicate in each animal and time point, totaling 126 bags/per animal. Filter bags plus samples were inserted into a washing laundry bag with a weight to allow for continual immersion within ruminal contents. Bags were placed into the rumen in the reverse order of incubation hours so that all bags were removed simultaneously for washing.

Once removed, bags were submerged for 15 min in saline solution with ice to stop the microbial activity and detach bacteria from the feed fraction. Then, bags were washed in a washing machine with running cold tap water until the rinsing water was clear. The 0 h bags were not incubated in the rumen but were rinsed in running water with the incubated bags. The bags were then oven-dried at 55 °C for 72 h [25]. After drying, the bags were individually weighed. Residues of each treatment were removed from bags and placed in a labeled plastic bag to obtain a sample of each treatment per animal/incubation time. Residual samples in the bags of different time points were used to estimate the parameters of ruminal degradation.

The DM and CP degradation profiles were estimated using the Ørskov and McDonald (1979) [26] asymptotic function:

Yt = A + B × (1 − e^−(kdt)^) (1)

where Yt is the fraction degraded in time ‘t’, g/kg; A is the water-soluble fraction, g/kg; B is the potentially degradable water-insoluble fraction, g/kg; kd is the degradation rate of fraction b, h^−1^; and t is time, h.

The effective degradability (ED, g/kg) of DM was calculated using the Denham et al. (1989) [27] model:

ED = A + [B × kd/(kd + kp) × e^−kpt^) (2)

where A is the water-soluble fraction, g/kg; B is the potentially degradable water-insoluble fraction, g/kg; kd is the degradation rate of fraction b, h^−1^; t is time, h; and kp is the rumen passage rate (k) of 0.074 h^−1^, obtained from the equation developed by NRC (2001) [28] for concentrates.

The RUP was calculated as follows:

RUP = B × [kp/(kp + kd)](3)

where B is the potential degradable water-insoluble fraction, g/kg; kd is the degradation rate, h^−1^; and kp is the passage rate, h^−1^.

### 2.3. Intestinal Digestibility Procedures

For in vitro trials, a system with four 4 L digestion vessels (TE-150, Tecnal Equipamentos Científicos, Piracicaba, SP, Brasil), equipped with a slow rotation and temperature controller was used in a 24 h fermentation batch. The three-step in vitro procedure proposed by Calsamiglia et al. (1995) [29] and modified by Gargallo et al. (2006) [30], was used to determine the ID of RUP. Briefly, 1000 mg (DM basis) of each ingredient from the timepoint of 12 h of the in situ incubation was weighed in duplicate into R510 bags (Ankom Technology, Macedon, NY, USA). Next, bags were sequentially incubated with constant rotation at 39 °C with pepsin solution (P-7000, Sigma, St. Louis, MO, USA) for 1 h and pancreatin solution (P-7545, Sigma) for 24 h. After incubation, bags were rinsed with tap water until effluent water remained clear. Then, samples were oven-dried at 60 °C for 48 h. Finally, residual samples in the bags were used to determine DM and N content.

### 2.4. Statistical Analysis

The DM and CP fractions, ED, RUP, and ID were first determined for each replication and compared using a completely randomized model design. Six contrasts tested differences across treatments:

- Control versus processing treatments;
- Effect of xylose;
- Linear effect of heating time;
- Quadratic effect of heating time;
- Interaction between xylose and heating time.

As heating time was not standardized across methods, all analyses were run separately for each processing method. All analyses were run using the PROC GLIMMIX of SAS (SAS on Demand, online version), and significance was declared when *p* < 0.05, and trends were declared when *p* < 0.10, as the critical probability level for type I error.

## 3. Results

### 3.1. Autoclave

**Figure 1:**
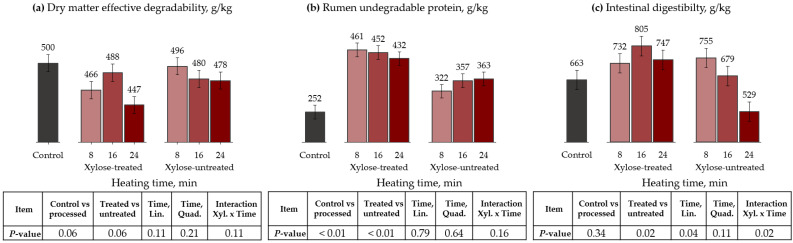
Effects of autoclave heating and xylose treatment on dry matter effective degradability (**a**), ED, rumen undegradable protein (**b**), RUP, and intestinal digestibility (**c**) of cottonseed meal. ED = A + [B × kd/(kd + kp) × e^−kt^] [28]; RUP = B × [kp/(kp + kd)], where A is the water-soluble fraction, g/kg; B is the potentially degradable water-insoluble fraction, g/kg; kd is the degradation rate of fraction b, h^−1^; t is time, h; and kp is the rumen passage rate of 0.074 h^−1^ [28].

The effects of time on autoclaving, with and without xylose, on ruminal variables are presented in Table 4 and Figure 1. For DM kinetics, the control had a greater fraction A (*p* < 0.01) and ED (trend, *p* = 0,06), compared to the remaining treatments. Furthermore, treatments without xylose tended to have greater ED (*p* = 0.06) than xylose-treated treatments. There was an interaction between xylose use and the different processing times for fraction B (*p* = 0.04). Heating time had a quadratic effect on fraction B for xylose-treated cottonseed meal, with the highest values reached at 16 min. The kd did not differ among treatments (*p* > 0.33).

Regarding protein kinetics, the control had greater fraction A and lower fraction B and RUP than processed treatments (*p* < 0.01). Xylose-treated samples had greater RUP than xylose-untreated samples (*p* < 0.01). Compared to the control, the RUP proportion increased by 83, 79, and 71% when 8, 16, and 24 min of the autoclave were applied to the xylose-treated cottonseed meal, respectively. Also, results showed interactions between the xylose use and processing times for fractions A (*p* < 0.01) and B (trend, *p* = 0.06), and kd (*p* = 0.02). Fraction A and kd linearly decreased, while fraction B linearly increased as the processing time increased, only for xylose-untreated cottonseed meal. Furthermore, heating time had a quadratic effect on kd for xylose-treated cottonseed meal, with the lowest value reached at 16 min. Xylose-treated cottonseed meal had greater ID than xylose-untreated (*p* = 0.02). Moreover, the interaction has shown that ID decreased as the processing time increased, only for treatments without xylose (*p* = 0.02).

### 3.2. Conventional Oven

**Figure 2:**
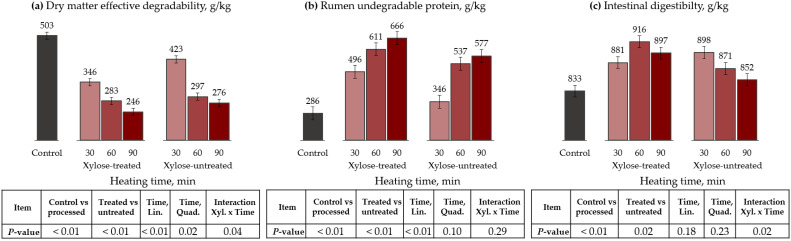
Effects of conventional-oven heating and xylose treatment on dry matter effective degradability (**a**), ED, rumen undegradable protein (**b**), RUP, and intestinal digestibility (**c**) of cottonseed meal. ED = A + [B × kd/(kd + kp) × e^-kt^] [28]; RUP = B × [kp/(kp + kd)], where A is the water-soluble fraction, g/kg; B is the potentially degradable water-insoluble fraction, g/kg; kd is the degradation rate of fraction b, h^−1^; t is time, h; and kp is the rumen passage rate of 0.074 h^−1^ [28].

For the conventional oven, ruminal degradation parameters of DM and CP, as well as protein ID, are presented in Table 5 and Figure 2. The control had greater DM fraction A (*p* < 0.01), kd (trend, *p* = 0.08), and ED (*p* < 0.01), compared to processed treatments. Furthermore, the interaction has shown that fraction A (only for xylose-untreated, *p* = 0.01) and ED (for both xylose-treated and -untreated, *p* = 0.04) linearly decreased as the processing time increased.

For CP kinetics, the control treatment also had greater A fraction (*p* = 0.03) and kd (trend, *p* = 0.10) than processed treatments. However, processing methods did not affect fraction B (*p* = 0.84). Nevertheless, the control treatment had lower RUP than processed treatments (*p* < 0.01). Furthermore, heating time linearly decreased fraction A (*p* = 0.03) and linearly increased RUP (*p* < 0.01). Moreover, xylose-treated cottonseed meal had a lower fraction A (*p* = 0.02) and greater RUP (*p* < 0.01), compared to xylose-untreated. Thus, the RUP proportion increased 73, 114, and 133% when 30, 60, and 90 min of heating were applied to xylose-treated cottonseed meal, respectively, compared to the control.

The processing methods affected ID, increasing this parameter compared to the control (*p* < 0.01). Furthermore, there was an interaction between xylose treatments and heating time, where increasing heating time linearly reduced the ID for xylose-untreated cottonseed meal (*p* = 0.02).

### 3.3. Microwave Oven

**Figure 3:**
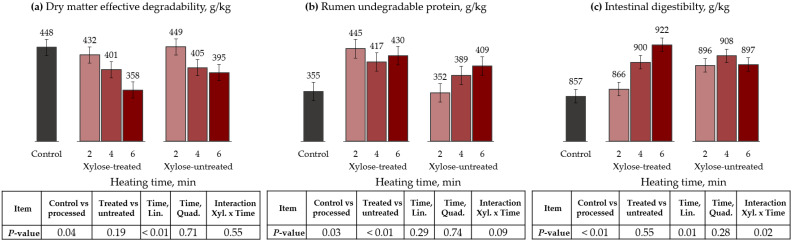
Effects of microwave-oven heating and xylose treatment on dry matter effective degradability (**a**), ED, rumen undegradable protein (**b**), RUP, and intestinal digestibility (**c**) of cottonseed meal. ED = A + [B × kd/(kd + kp) × e^−kt^] [28]; RUP = B × [kp/(kp + kd)], where A is the water-soluble fraction, g/kg; B is the potentially degradable water-insoluble fraction, g/kg; kd is the degradation rate of fraction b, h^−1^; t is time, h; and kp is the rumen passage rate of 0.074 h^−1^ [28].

Regarding DM degradation kinetics, the control had a greater fraction A (*p* = 0.04) and ED (*p* = 0.04), and lower fraction B (*p* = 0.03) than processed treatments (Table 6 and Figure 3). Furthermore, the time of microwave heating linearly reduced the fraction A and ED of processed treatments (*p* < 0.01).

For CP kinetics, fraction B (trend, *p* = 0.09) and RUP was lower (*p* = 0.03) for control than for processed cottonseed meals. Also, there was an interaction between the xylose’s use and processing times for fractions A (*p* = 0.04) and B (trend, *p* = 0.06). Thus, heating time decreased fraction A and increased fraction B only for xylose-treated cottonseed meal. On the other hand, RUP tended to increase as the heating time increased, only for xylose-untreated cottonseed meal (*p* = 0.09). Nevertheless, the RUP proportion increased 25, 17, and 21% compared to the control when 2, 4, and 6 min of heating were applied to xylose-treated cottonseed meal, respectively.

The ID was also lower for control than the processed treatments (*p* < 0.01). Moreover, there was an interaction between xylose treatments and heating time, where increasing heating time linearly increased the ID for xylose-treated cottonseed meal (*p* = 0.02).

## 4. Discussion

This study addresses different methods to increase the RUP of cottonseed meal, making it a possible alternative to supply the high RUP requirements of high-performance beef cattle. Thus, to our knowledge, the effects of heating processes (autoclave, conventional, and microwave ovens) plus xylose on protecting cottonseed meal protein from ruminal fermentation remains unclear. As these techniques may result in changes in protein solubility [9], we hypothesize that the DM and CP ruminal kinetic parameters of processed cottonseed meals may change by application of heat and xylose. Indeed, our results indicate a decrease in the soluble fraction of DM for all processing methods, compared to unprocessed cottonseed meal. Moreover, compared to the control, autoclaves and conventional ovens also decreased the soluble fraction of protein. Furthermore, the conventional oven reduced the percentage of dry matter and protein degraded in the rumen over time, compared to the control. Also, the fraction A of protein was decreased as heating time increased. These results are expected, once soluble components are more affected by heating processes, which reduce their availability for microbial enzymatic degradation of protein [31,32]. Compared to the control, this heating was also strong enough to increase the protein fraction B of cottonseed meal processed in both autoclave and microwave ovens. Thus, our results suggest that these techniques changed ruminal nutrient kinetics and increased RUP.

As expected, the changes in rumen kinetics were reflected in the lower ED and greater RUP for all processing methods compared to untreated cottonseed meal. Hence, heat may expose the hydrophobic components and consequently decrease protein solubility, and in addition to the binding among sugar and amino acids, the rumen degradation of this nutrient is reduced [33]. Thus, since there is high complexity in protein protection mechanisms against rumen degradation [34], heating makes proteins more resistant to microbial enzymes [35]. Others also have observed a RUP increase caused by the heating process on different feeds, such as canola meal (conventional and microwave ovens) [18,36], dry corn (microwave) [19], linseed (microwave) [37], and soybean meal (toasting), [38]. However, it is essential to investigate whether these processing methods impact protein ID.

Xylose treatment of protein sources can strengthen the binding among carbohydrates and amino acids when associated with heat [34,35]. Thus, we expected that DM and CP kinetics would be changed for xylose-treated cottonseed meal. As expected, xylose-treated groups had lower ED and greater RUP than untreated cottonseed meals. Furthermore, these changes were more effective for cottonseed heated in a conventional oven, where xylose-treated cottonseed meal had greater DM and CP soluble fractions than the xylose-untreated group. Moreover, for this processing method, there was an association between sugar and time, resulting in less DM-soluble-fraction protection as heating time was increased. However, the same effect was not observed for protein kinetics, which suggests that heating in a conventional oven may have a protective impact on other nutrients. Nevertheless, our results showed an additive effect of xylose and heat on protein protection from rumen fermentation. Thus, it is interesting to see if these changes in the ruminal degradability of processed cottonseed meals may also affect their ID, since protein could be unavailable for further compartments’ digestion if these processing methods are overstated [12,15].

Because mechanisms underlying heating feed may impact amino-acid availability through post-rumen compartments [39], we have assessed the protein ID of cottonseed meal with different processing methods. Interestingly, our results suggest a greater feed intestine digestion for processed cottonseed meals. Thus, the processes regarding post-rumen digestion and absorption of these ingredients should be further studied. Regarding evaluation of the control versus other treatments, the greater ID observed for conventional and microwave ovens, compared to the control, are exciting and promising results. As observed here, others found an increased ID of heat-treated hempseed cake [40]. These authors suggested that the antinutritional components (such as tannins and trypsin inhibitors) may be decreased by the heating process, which affected the protein ID [41]. Furthermore, heat processing may bind protein with gossypol (a phenolic compound found in cottonseed), inactivating its toxicity [4,42,43,44]. Thus, we speculate that the underlying mechanisms regarding heating effects on gossypol might also affect protein protection. Thus, these mechanisms should be the focus of a further investigation. Sadeghi and Shawrang (2007) [3] also observed greater ID of microwave heated (for 4 min) cottonseed meal and reported that this result was due to protein denaturation. Indeed, according to Murray et al., 2003 [45], microwave irradiation might unfold protein structure and expose sites of action used by pancreatic proteases, which may increase ID. Moreover, Pena et al., (1986) [46] observed positive effects of conventional heating of whole cottonseed on total amino-acid flow to the intestine of Holstein cows. Other researchers did not observe total tract digestibility effects but roasted cottonseed meal tended to improve milk protein and production in dairy cattle [47]. Finally, the greater ID for xylose-treated than for xylose-untreated groups in the autoclave and conventional-oven trials suggests that this sugar may catalyze the improvement of protein ID.

Therefore, our results lead us to conclude that the RUP content may be increased by heating processes plus xylose addition. These three tested techniques have different ways to apply heat: for the conventional oven method, feedstuff heating goes from the surface to the interior, which requires more processing time and results in an uneven heat effect on protein structure [37]; the autoclave uses vapor heating plus pressure, which may allow a more uniform heating distribution throughout the sample [48]; and the microwave heating process absorbs the electromagnetic energy produced by a magnetron to heat uniformly and rapidly [49]. However, caution must be taken with the time and intensity of heat exposure because overheating may affect amino-acid availability for intestinal absorption. In summary, our results suggested some potential treatments to be used for each heating process. Regarding the autoclave, we highlight the xylose-treated cottonseed meal with 8 and 16 min heating, which would have 102 and 118% more RUP digested in the intestine than non-processed cottonseed meal. For a conventional oven, 90 min of heating for xylose-treated cottonseed meal was the most promising treatment under the conditions of the present study. It could increase the intestine-digested RUP by 151% compared to the control. Concerning microwave ovens, the best treatments were: xylose-treated cottonseed meals with 2, 4, and 6 min heating, which increased the intestine digestible RUP up to 27, 23, and 30%, respectively. The next steps are to conduct studies to evaluate the rumen fermentation parameters of these ingredients.

## 5. Conclusions

Our results suggest that the evaluated heating processing methods (autoclave, conventional, and microwave ovens), associated with xylose treatment could modulate the cottonseed meal characteristics by improving protein protection from ruminal fermentation and increasing RUP. Therefore, the best treatments under these experimental conditions were: autoclave, xylose-treated cottonseed meal with 8 and 16 min of heating; conventional oven, 90 min heating for xylose-treated cottonseed meal; and for the microwave oven, xylose-treated cottonseed meals with 2, 4, and 6 min of heating. However, studies are necessary to confirm the results found herein and evaluate the effects of these processed feeds on ruminal fermentation parameters, animal performance, and economic viability.

## Figures and Tables

**Table 1:** Chemical composition of experimental ingredients of Experiment 1—autoclave.

Item	Control	Xylose-Treated			Xylose-Untreated		
		8	16	24	8	16	24
Dry matter, g/kg	910	899	899	900	904	902	902
Organic matter, g/kg DM	937	94.3	943	943	936	938	948
Crude protein, g/kg DM	543	449	474	471	501	490	482
Neutral detergent fiber, g/kg DM	169	351	360	358	276	305	289

**Table 2:** Chemical composition of experimental ingredients of Experiment 2—conventional oven.

Item	Control	Xylose-Treated			Xylose-Untreated		
		30	60	90	30	60	90
Dry matter, g/kg	910	920	923	923	920	921	932
Organic matter, g/kg DM	937	923	935	941	939	936	937
Crude protein, g/kg DM	543	524	508	472	535	558	509
Neutral detergent fiber, g/kg DM	169	304	446	471	341	374	386

**Table 3:** Chemical composition of experimental ingredients of Experiment 3—microwave oven.

Item	Control	Xylose-Treated			Xylose-Untreated		
		2	4	6	2	4	6
Dry matter, g/kg	910	905	921	920	909	912	918
Organic matter, g/kg DM	937	938	935	929	932	926	939
Crude protein, g/kg DM	543	462	534	581	534	549	533
Neutral detergent fiber, g/kg DM	169	394	376	323	367	238	326

**Table 4:** Effects of autoclave and xylose inclusion on rumen degradation parameters of cottonseed meal.

Item ^1^	Control	Xylose-Treated ^2^			Xylose-Untreated ^2^			SEM	*p*-Value				
		8	16	24	8	16	24		Control × Processed	Xyl.-Treated × -Untreated	Time, Lin.	Time, Quad.	Interaction Xyl. × Time
**Dry Matter**													
A, g/kg	319	246	230	235	261	250	240	12.9	<0.01	0.24	0.24	0.61	0.86
B, g/kg	475	459	605	500	505	494	478	34.5	0.41	0.32	0.84	0.05	0.04
kd, h^−1^	0.051	0.072	0.060	0.053	0.065	0.065	0.073	0.01	0.33	0.56	0.66	0.76	0.64
**Crude Protein**													
A, g/kg	552	104	198	217	396	287	164	45.8	<0.01	0.01	0.22	0.58	<0.01
B, g/kg	380	636	686	597	486	602	679	55.6	<0.01	0.29	0.19	0.38	0.06
kd, h^−1^	0.077	0.189	0.080	0.107	0.102	0.107	0.179	0.03	0.15	0.87	0.94	0.08	0.02

^1^ A, water-soluble fraction; B, potentially degradable water-insoluble fraction; kd, degradation rate of fraction B; SEM, standard error of the mean. ^2^ Autoclave heating times: 8, 16, and 24 min (127 °C and 117 kpa of pressure).

**Table 5:** Effects of conventional-oven heating and xylose inclusion on rumen degradation parameters of cottonseed meal.

Item ^1^	Control	Xylose-Treated ^2^			Xylose-Untreated ^2^			SEM	*p*-Value				
		30	60	90	30	60	90		Control × Processed	Xyl.-Treated × -Untreated	Time, Lin.	Time, Quad.	Interaction Xyl. × Time
**Dry Matter**													
A, g/kg	292	196	183	178	246	184	182	7.30	< 0.01	0.02	< 0.01	0.03	0.01
B, g/kg	517	611	466	632	486	831	818	294	0.71	0.57	0.57	0.97	0.43
kd, h^−1^	0.057	0.031	0.030	0.016	0.044	0.014	0.017	0.015	0.08	0.92	0.19	0.69	0.36
**Crude Protein**													
A, g/kg	475	341	291	233	476	372	342	45.3	0.03	0.02	0.03	0.68	0.57
B, g/kg	485	537	579	252	443	693	691	213	0.84	0.41	0.94	0.43	0.64
kd, h^−1^	0.074	0.051	0.026	0.049	0.048	0.015	0.016	0.019	0.10	0.35	0.41	0.27	0.85

^1^ A, water-soluble fraction; B, potentially degradable water-insoluble fraction; kd, degradation rate of fraction B; SEM, standard error of the mean. ^2^ Heating times: 30, 60, and 90 min in a conventional oven (150 °C).

**Table 6:** Effects of microwave-oven heating and xylose inclusion on rumen degradation parameters of cottonseed meal.

Item ^1^	Control	Xylose-Treated ^2^			Xylose-Untreated ^2^			SEM	*p*-Value				
		2	4	6	2	4	6		Control × Processed	Xyl.-Treated × -Untreated	Time, Lin.	Time, Quad.	Interaction Xyl. × Time
**Dry Matter**													
A, g/kg	199	185	153	134	195	173	153	14.1	0.04	0.17	< 0.01	0.77	0.74
B, g/kg	553	597	606	632	621	620	660	25.9	0.03	0.31	0.17	0.53	0.94
kd, h^−1^	0.060	0.052	0.050	0.041	0.051	0.045	0.043	0.008	0.15	0.88	0.24	0.87	0.86
**Crude Protein**													
A, g/kg	365	223	302	355	309	345	269	36.7	0.13	0.64	0.23	0.30	0.04
B, g/kg	582	729	637	613	647	616	685	37.1	0.09	0.74	0.31	0.21	0.06
kd, h^−1^	0.067	0.062	0.060	0.042	0.085	0.057	0.065	0.015	0.78	0.23	0.20	0.70	0.97

^1^ A, water-soluble fraction; B, potentially degradable water-insoluble fraction; kd, degradation rate of fraction B; SEM, standard error of the mean. ^2^ Heating times: 2, 4, and 6 min in microwave oven (1000 W, full power).

## Data Availability

The data presented in this study are available on request from the corresponding author.
